# Editorial: Genetics, genomics and climatic adaptation in tropical conditions

**DOI:** 10.3389/fgene.2024.1445498

**Published:** 2024-07-09

**Authors:** Johanna Ramírez-Díaz, Arianna Manunza, Tania Bobbo, Juliana Petrini, Juan Carlos Rincón-Florez

**Affiliations:** ^1^ National Research Council, Institute of Agricultural Biology and Biotechnology, Milano, Italy; ^2^ Institute for Biomedical Technologies, National Research Council (CNR), Milano, Italy; ^3^ Clinica do Leite, Sao Paulo, Brazil; ^4^ Departament of Animal Science, National University of Colombia, Valle del Cauca, Colombia

**Keywords:** ANGR, animal breeding, environmental condition, genetic adaptation, climate change

Scientific evidence demonstrates that environmental pressures can shape the animal genomes. Climatic factors like temperature, humidity, precipitation, wind velocity, altitude and atmospheric pressure play a significant role in determining the distribution and performance of species (Salvian et al., 2023). In tropical regions, livestock breeds are usually well adapted to harsh environmental conditions. Nevertheless, the accelerated climate change impacts water and feed availability, disease resistance, animal welfare, production levels and reproductive performance on a global scale.

Strategies that include genetic diversity assessment (Manunza et al., 2023), identification and strengthening of local breeds, genetic selection, crossbreeding, as well as assessment of spatial genetic patterns (Ramirez-Diaz J et al., 2023) and their association with selection signatures in the genome could improve genetic gain and offer new opportunities for population conservation. Multi-omics approaches coupled with advanced statistical and software tools can deepen the understanding about the genetic bases for mitigating climate change.

Genetic characterization methods provide insights into the genetic basis of breed formation and reveal variations or similarities between populations. This helps to identify the uniqueness of a population and guides future breeding or conservation initiatives. For instance, Cendron et al. investigated the diversity and the genetic background of the Siboney creole cattle using information of 213 bovine worldwide populations and 16,102 common SNP (Bovine 100K BeadChip). The admixture analysis evidenced that Siboney cattle shares the genetic background with *indicus* cattle from Asia, Africa and America, and diversity indices showed high levels of diversity associated with the recent history of the breed (HE: 0.404 ± 0.103; HO: 0.409 ± 0.404) and low levels of inbreeding (FHOM: −0.013 ± 0.033 and FROH: 0.004 ± 0.002). The genomic locations of ROH were associated with genes potentially under selection and related to growth (BTA11: *PPM1B*; BTA26: *ADD3*, BTA14: *NSMAF*), carcass (BTA26: *RAD21*) and milk traits (BTA26: *XPNPEP1*).

Heat stress is a physiological response of the animal when it is unable to dissipate heat efficiently. In that situation, the goal is to obtain animals that are able to balance the thermogenesis and heat dissipation, maintaining their performance under heat conditions. Considering that there is genetic variability for heat stress tolerance, genetic selection can be a tool to mitigate the heat effects on livestock populations. Habimana et al. conducted a comprehensive review of heat stress indicators and genetic selection methods in dairy cattle, highlighting negative implications such as the decline in milk yield, alteration of milk composition, adverse physiological reactions, reduced feed intake, and low reproduction rates. They discussed the use of Random Regression models using broken line and Legendre polynomials functions as well as Reaction Norm models, and a practical review about the genes responsible for heat tolerance was presented. In addition, effects of breed composition on skin histological traits in a multibreed population was evaluated by Mateescu et al. based on least square means. Such analysis revealed significant effects (*p* < 0.05) of the breed group on Epidermis (thickness and length), Sweat Gland (depth, Length and area), as well sebaceous gland (area and number) suggesting that phenotypic variation of the skin can be explorer in selective improvement.

Environmental factors, and especially heat stress, are known to adversely affect milk synthesis in dairy cattle. Indeed, milk production and the growth of cow mammary epithelial cells are influenced by several factors, including amino acids, glucose, hormones and environmental stressors. Amino acids, beyond serving as the building blocks for protein synthesis, are essential in regulating cell proliferation and casein synthesis in mammalian epithelial cells. Li et al. focused their review on the mammalian target of rapamycin complex 1 (mTORC1) pathway, which is recognized as the key signalling pathway that regulates cell proliferation and the synthesis of milk protein and fat in cow mammary epithelial cells in response to amino acids and heat stress. In particular, the authors explored the main regulatory genes, the effects of amino acids and heat stress on milk production, and the regulation of the mTORC signalling pathway in cow mammary epithelial cells.
